# Efficacy of Yoga in Treating Positive and Negative Symptoms of Schizophrenia and Enhancing Social Functioning: A Systematic Review and Meta-Analysis of Randomized Controlled Trials

**DOI:** 10.1192/j.eurpsy.2025.343

**Published:** 2025-08-26

**Authors:** M. L. Geremias, A. Victoria de Vasconcelos, A. L. Balduino de Souza, I. Borja De Oliveira, I. Santos Raposo Andrade, V. M. Soares Campos, D. Soler Lopes

**Affiliations:** 1 Medicine, University of Joinville Region (UNIVILLE), Joinville; 2 Medicine, Afya College of Medical Sciences of Santa Inês, Santa Ines; 3 Medicine, Evangelical University of Goias, Anapolis; 4 Medicine, University of São Paulo, São Paulo; 5 Medicine, Pontifícia Universidade Católica de Campinas, Campinas; 6 Medicine, Federal University of Santa Catarina (UFSC), Florianópolis, Brazil; 7 Medicine, Lancaster University, Lancaster, United Kingdom

## Abstract

**Introduction:**

Schizophrenia is a chronic mental disorder marked by positive symptoms such as hallucinations and delusions, and negative symptoms such as social withdrawal and apathy. While traditional pharmacological treatments effectively manage positive symptoms, they often fall short in addressing negative symptoms and social functioning. Yoga has emerged as a complementary therapy that may help improve both. However, its overall impact remains uncertain.

**Objectives:**

This review aims to synthesize evidence on yoga’s effectiveness in reducing positive and negative symptoms of schizophrenia and enhancing social functioning.

**Methods:**

A systematic search was conducted in Scopus, Web of Science, and PsycINFO in September 2024, following the Preferred Reporting Items for Systematic Reviews and Meta-Analyses (PRISMA) guidelines. Included studies were peer-reviewed randomized controlled trials (RCTs) assessing yoga’s effects on symptom severity and social functioning in schizophrenia. A fixed-effects or random-effects model was applied, with subgroup analyses performed. Standard mean differences (SMDs) and mean differences (MDs) were used for effect size estimation.

**Results:**

Sixteen RCTs were included, involving 862 participants. Yoga (n = 393) was compared to three control groups: treatment-as-usual (n = 152), other physical activities (n = 124), and waitlist (n = 193). Yoga significantly reduced overall symptom severity relative to control, as shown by a decrease in PANSS scores (MD = -6.61, 95% CI -13.21 to -0.0, p = 0.05, I² = 82%). It significantly improved positive symptoms relative to waitlist (SMD = -0.87 [-1.70, -0.03]) and treatment-as-usual (SMD = -0.65 [-1.21, -0.09]), with effects comparable to those observed with physical activity (MD = -1.30 [-3.09, 0.49]). There were no significant effects on negative symptoms, with SMDs of -1.59 [-4.18, 1.01] for the waitlist and -1.16 [-2.41, 0.09] for treatment-as-usual, and a MD of -1.32 [-3.60, 0.97] when compared to physical activity outcomes. Additionally, yoga did not significantly impact social functioning, showing SMDs of -0.44 [-1.56, 0.68] for the waitlist and -0.44 [-2.29, 1.41] for treatment-as-usual, and a MD of 2.30 [-0.74, 5.34] for physical activity.

**Conclusions:**

This review shows that yoga is effective in reducing overall symptom severity compared to controls and improves positive symptoms relative to treatment-as-usual or waitlist, but not compared to physical activity. There were no significant differences in alleviating negative symptoms or enhancing social functioning. These findings suggest yoga may be a promising adjunctive treatment for positive symptoms of schizophrenia, especially when traditional treatments are insufficient. Further high-quality RCTs with standardized protocols are needed to confirm these results and establish optimal treatment parameters.

**Disclosure of Interest:**

None Declared

